# Mycorrhizae and grapevines: the known unknowns of their interaction for wine growers’ challenges

**DOI:** 10.1093/jxb/eraf081

**Published:** 2025-03-11

**Authors:** Maider Velaz, Luis Gonzaga Santesteban, Nazareth Torres

**Affiliations:** Dept. of Agronomy, Biotechnology and Food Science, Public University of Navarre, Campus Arrosadia, 31006 Pamplona, Navarra, Spain; Institute for Multidisciplinary Research in Applied Biology (IMAB-UPNA), Public University of Navarre, Campus Arrosadia, 31006 Pamplona, Navarra, Spain; Dept. of Agronomy, Biotechnology and Food Science, Public University of Navarre, Campus Arrosadia, 31006 Pamplona, Navarra, Spain; Institute for Multidisciplinary Research in Applied Biology (IMAB-UPNA), Public University of Navarre, Campus Arrosadia, 31006 Pamplona, Navarra, Spain; Dept. of Agronomy, Biotechnology and Food Science, Public University of Navarre, Campus Arrosadia, 31006 Pamplona, Navarra, Spain; Institute for Multidisciplinary Research in Applied Biology (IMAB-UPNA), Public University of Navarre, Campus Arrosadia, 31006 Pamplona, Navarra, Spain; University of Warwick, UK

**Keywords:** Arbuscular mycorrhizal fungi, grapevine, microbiome, nursery, rootstock, strigolactone, vineyard

## Abstract

Arbuscular mycorrhizal fungi (AMF) play an important role in grapevine production systems. However, little is known about how this relationship is achieved in the nursery and how soil management might modify it and its derived benefits. Here, we review the current knowledge on the establishment of grapevine–AMF relationships from the nursery to the field, the main factors that affect the effectiveness of the symbiosis, the potential role of AMF as biostimulants in grapevine production systems, and the future perspectives of their use in the current context of climate change. The process of establishing mycorrhizal symbiosis is complex, and the molecular dialogue between the plant roots and the fungus is still not yet fully understood. During vine plant production, rooting occurs in nurseries, where spontaneous symbiosis can be generated. The effectiveness of mycorrhizal symbiosis appears to depend not only on the identity of the fungus but also on the diversity of the vine material and soil management. Finally, the use of AMF as biostimulants might be an effective strategy with which to face the new climatic scenario, but further research dealing with the application of AMF inocula and the protection of native cohorts should be conducted.

## Teaser

A review of establishment of grapevine–arbuscular mycorrhizal fungi interaction from the nursery to the vineyard, including the biotic and abiotic factors affecting them and their potential use as biostimulants.

## Introduction

The roots of cultivated grapevine (*Vitis vinifera* L.) are known to interact with arbuscular mycorrhizal fungi (AMF) in a significant way. There is abundant evidence that symbiosis with AMF can ameliorate plant performance under environmental stresses related to climate change (reviewed in Torres *et al.*, 2018a), although their presence in the soil does not always provide benefits to the host. However, the role of AMF in vineyards is not sufficiently well understood. In this review, we present current knowledge of the spontaneous establishment of the grapevine–AMF relationship from the nursery to the field, the main factors affecting the effectiveness of the symbiosis, and the potential role of AMF as biostimulants in grapevine production systems, and we discuss future perspectives of using AMF in the context of climate change.

Grapevine has been cultivated for more than 7000 years and on every continent except for Antarctica. The cultivated surface globally accounts for >7.4 million hectares, 107.5 million tons of berry production, and 262 million hectoliters of wine (OIV, 2023). Due to its global economic importance, the climatic diversity of the producing regions, its sensitivity to changes in cultivation practices, and the large number of studies (from genomics to production practices), grapevine has emerged as a model plant for perennial fruit crop species (Gambetta *et al*., 2020) and an exceptional subject for studying soil–plant continuum interactions. The concept of ‘terroir’, sometimes controversial, mainly used to refer to wine grapes, highlights that different factors such as the cultivated variety, the location of the vineyard (climate and soil), and vineyard management link the agricultural product (quality, taste, style) to its origin (Van Leeuwen and Seguin, 2006).

In this regard, the term ‘microbial terroir’ has been recently coined to emphasize the potential role of the microbiome associated with grapevine organs (including berries and roots) (Bokulich *et al*., 2013; Zarraonaindia *et al*., 2015; Lazcano *et al*., 2020) in the process of solving the scientific puzzle of the relevance of terroir in wine typicity. It is well established that vineyard microbiomes are shaped by vineyard geography and management (Bokulich *et al*., 2016; Miura *et al*., 2017; Coller *et al*., 2019; Torres *et al*., 2021b; Teixeira *et al*., 2024). In fact, Zarraonaindia *et al*. (2015) reported that, even within the same region, there was a significant variation in the community structure in both soil and vine microbiomes, suggesting a differential selection by the vines from the soil microbial seed bank.

In addition, these interactions can, directly or indirectly, modify plant behavior, so there is a challenge to understand grapevine not as an independent organism, but as a ‘holobiont’ (Bettenfeld *et al*., 2022). This concept emphasizes the ubiquitous interaction between the plant and the microbes involved within all parts of the plant that firstly occurred when ancestral plants settled on land 450 million years ago (Hassani *et al*., 2018). Microbes colonize the rhizosphere (surface of belowground plant parts; roots), phyllosphere (surface of aboveground plant parts; leaves), and endosphere (within plant tissues) (Hassani *et al*., 2018; Bettenfeld *et al*., 2022). Berry colonizing microorganisms could migrate from roots, dust rain splashes, or people during harvesting and other management events (Compant *et al*., 2011; Gilbert *et al*., 2014), and endophytic bacterial microbiota found in grapevine shoot xylem seem to depend on the cultivar, the growing region and the shoot growth stage (Hamaoka *et al*., 2022).

Among grapevine colonizing microorganisms, arbuscular mycorrhizal fungi (AMF) stand out, given that they can establish a mutually beneficial symbiosis with grapevine roots and constitute a direct link between the soil and the vine. Therefore, the natural symbiosis between grapevines and species belonging to the *Glomeromycetes* class (AMF) found in vineyards provides several ecosystem services and contributes to the ‘terroir’ (Trouvelot *et al*., 2015). Trouvelot *et al*. (2015) highlighted that this symbiosis increased grapevine growth and improved nutritional status through better access to soil nutrients and activation of the plant transport proteins. Nevertheless, understanding what being a holobiont means could be more complex and might require including the plant, its associated mycobiota, and the microbiota of it (Bonfante *et al.*, 2019). Thus, some studies have tested whether AMF species from diverse evolutionary lineages and origins possessed endobacteria (Naumann *et al.*, 2010; Lastovetsky *et al.*, 2024) and mycoviruses (Ikeda *et al.*, 2012). Despite endobacteria representing a metabolic cost to the AMF, which must provide nutrients for their basic metabolism, the widespread occurrence of endosymbiotic bacteria among AMF suggests that they assist their host with some benefits (Bonfante *et al.*, 2001). Similarly, Ikeda *et al.* (2012) demonstrated that some mycoviruses had evolved under unique selection pressures and actively contributed to the symbiosis. The role of these endobacteria in the symbiosis remains unresolved, and different hypotheses have been proposed. A recent study highlighted that the presence of a known endobacterium, *Candidatus* Moeniiplasma Glomeromycotorum, can limit the development of other endobacteria that exploit the lipid-rich AMF spores (Lastovetsky *et al.*, 2024). Likewise, it was reported that the symbiosis between AMF and endobacteria increases the fitness of a mycorrhizal fungus, raising its bioenergetic capacity and acting as a potential driver for priming the fungal innate immunity (Salvioli *et al.*, 2016).

## The molecular dialogue between plant roots and arbuscular mycorrhizal fungi during symbiosis establishment

**Fig. 1.**
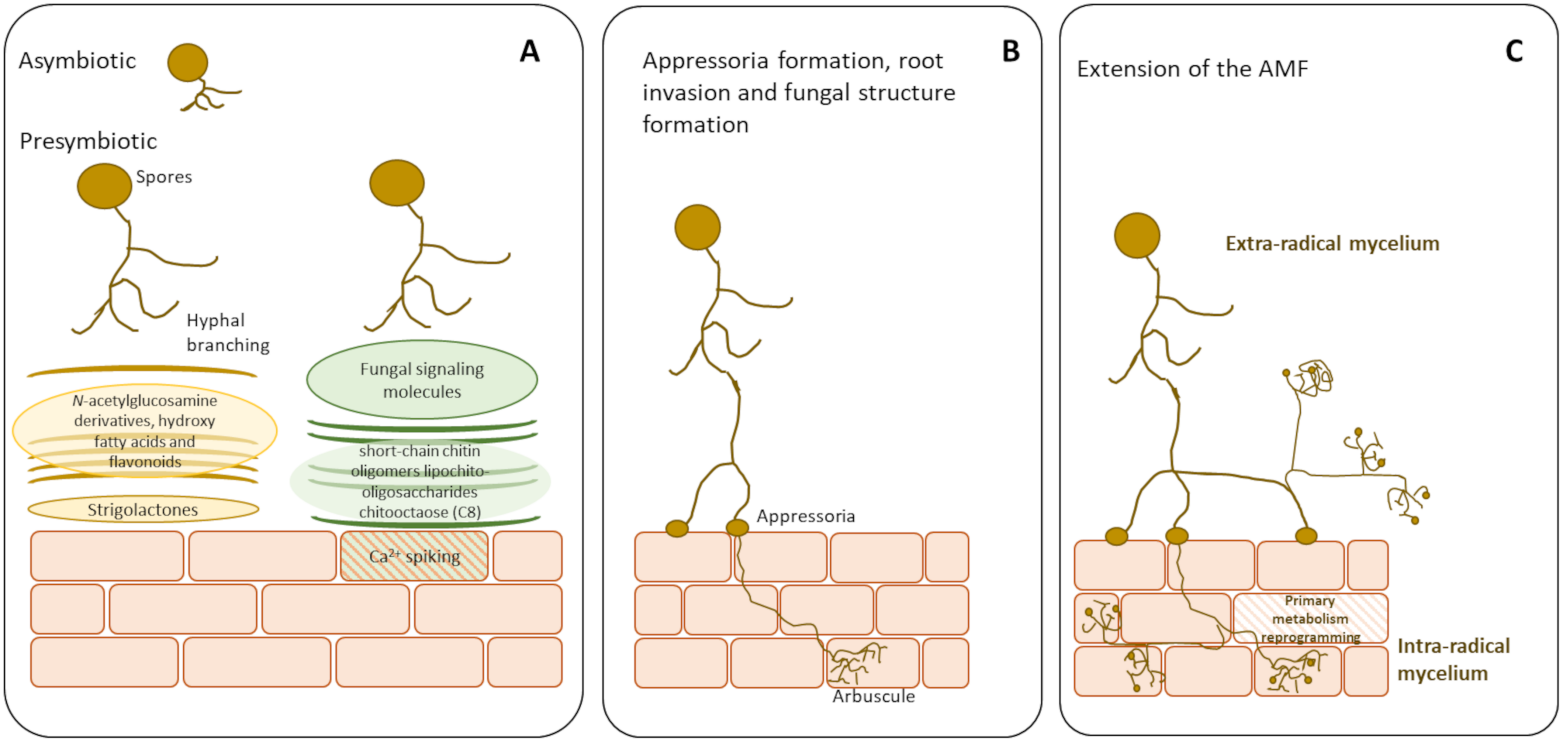
Diagram of the establishment of the mycorrhizal symbiosis. (A) Asymbiotic and presymbiotic stages, (B) appressoria formation, root invasion, and fungal structures formation, and (C) ramification of the arbuscular mycorrhizal fungi (AMF).

The process of mycorrhization and the molecular dialogue that occur during its establishment are complex, involving several events (Fig. 1). Within this process, the most crucial event is the attachment and penetration of the plant surface, through a process in which *Glomeromycota* fungi must transit from an asymbiotic to a symbiotic stage. While still asymbiotic, AMF spores germinate after elicitation by exudates from the roots, leading to extensive hyphal branching (Giovannetti *et al*., 1993; Akiyama *et al*., 2005; Fiorilli *et al.*, 2024), which is essential to reach the grapevine roots (Fig. 1A). Strigolactones are apocarotenoids synthesized and exuded by roots into the rhizosphere as messages to mediate between the plant and possible symbionts, as well as to intervene in plant-to-plant interactions (Wheeldon *et al*., 2022; Clark and Bennett, 2023). They also promote the elongation of root hairs and the growth of primary roots, and inhibit the formation of adventitious and lateral roots (reviewed by Al-Babili and Bouwmeester, 2015). Furthermore, it has been reported that strigolactones modulate the microbiome associated with the roots of herbaceous plants such as rice (Nasir *et al*., 2019), soybean (Liu *et al*., 2020). and Arabidopsis (Carvalhais *et al*., 2019), but little is yet known about their effect on woody plants. In fact, a recent study described the role of vitislactone, a non-canonical strigolactone that has never been identified in another species, in stimulating beneficial root–microbe interactions in grapevines (Lailheugue *et al*., 2023). Without the presence of the host plant, AMF spores may germinate, but only limited hyphal growth will occur up to 30 d after germination, and then, if a host plant is not found, growth stops (Harrier, 2001). In addition to strigolactones, other root-exudate molecules such as *N*-acetylglucosamine derivatives, hydroxy fatty acids, and flavonoids may induce AMF branching and concomitant responses (reviewed by Steinkellner *et al*., 2007; Haichar *et al*., 2014). Then, the fungal production of signaling molecules activate the symbiosis signaling pathway and trigger calcium spiking in the rhizodermal cells (Bonfante and Desirò, 2015) (Fig. 1A). Once recognition between the plant and the fungus occurs, appressorial structures differentiate through a process that implies different signals exuded by the plant host, such as cutin monomers (Sharma and Gautam, 2019) and ethylene (Deising *et al*., 2000; Fig. 1B). Appressoria are specialized cells or adhesion structures produced by fungi, from which a penetration peg emerges and pierces the host tissues (Chethana *et al*., 2021). At this stage, the fungus penetrates the root epidermis and starts the intracellular invasion of the root, leading to symbiotic colonization (Fig. 1C). This phase is characterized by the formation of intracellular and intercellular hyphae, vesicles, and arbuscules constituting the intra-radical mycelium (IRM), and a later extensive growth of a sporulative and highly branched extra-radical mycelium (ERM). In this phase, lipid-rich vesicles are accumulated until the ERM is able to form asexual chlamydospores, finalizing the fungal life cycle (Smith and Read, 2008). Once the relationship is established, AMF trigger major reprogramming of primary metabolism in grapevine roots, especially sugar and fatty acid metabolism (Goddard *et al*., 2021; Sportes *et al.*, 2023). Thus, in this mutualistic association, the plant supplies the fungus with carbohydrates and lipids, and fungal colonization increases the root absorption surface, improving plant access to water and minerals. In fact, Goddard *et al.* (2021) found that unsaturated fatty acids were enhanced in roots and leaves of mycorrhized ungrafted vines, together with higher levels of salicylic acid and jasmonic acid in leaves and pathogenesis-related protein accumulation in roots. This, jointly with the higher concentrations of active forms of stilbenoids found in mycorrhizal plants (Nerva *et al*., 2023), can potentially confer mycorrhiza-induced resistance (MIR) in grapevine, a response through which mycorrhizal plants tolerate potential biotic and abiotic stresses better (Bettenfeld *et al*., 2022) and consequently maintain the functionality and persistence of the relationship. In this context, small RNAs have been proposed to be key factors for MIR in this molecular dialogue; small RNA effectors transferred between AMF and plants facilitate cross-kingdom RNA interference, suppressing plant defense mechanisms during AMF colonization (Fiorilli *et al.*, 2024).

Within this molecular dialogue, it is important to stress that fungal mycelia link two or more plants, either of the same or different species, into mycorrhizal networks. As a result, these networks can integrate various plant and fungal species into a dynamic, adaptive system that interacts, responds to feedback, and adjusts accordingly (Gilbert and Johnson, 2017). Thus, mycorrhizal networks offer inter-plant communication by transferring nutrients, stress signals, and allelochemicals (Gilbert and Johnson, 2017; Boyno and Demir, 2022). Many of these signals travel rapidly—within hours or days—both within and between plants. For instance, carbon (C) and nitrogen (N) are transferred from donor plants to the fungal mycelium within 1–2 d, and then from the mycelium to the shoots of neighboring plants within 3 d (reviewed by Boyno and Demir, 2022). Moreover, stress signals are transmitted through mycorrhizal networks at a faster rate than C, water, or nutrients, moving more swiftly from damaged plants to healthy ones. For example, stress signals triggered by herbivory or fungal invasion in donor plants have been shown to be up-regulated in neighboring, undamaged plants via mycorrhizal networks within just 6–24 h (reviewed by Boyno and Demir, 2022).

## The nursery, where mycorrhization may start

**Fig. 2.**
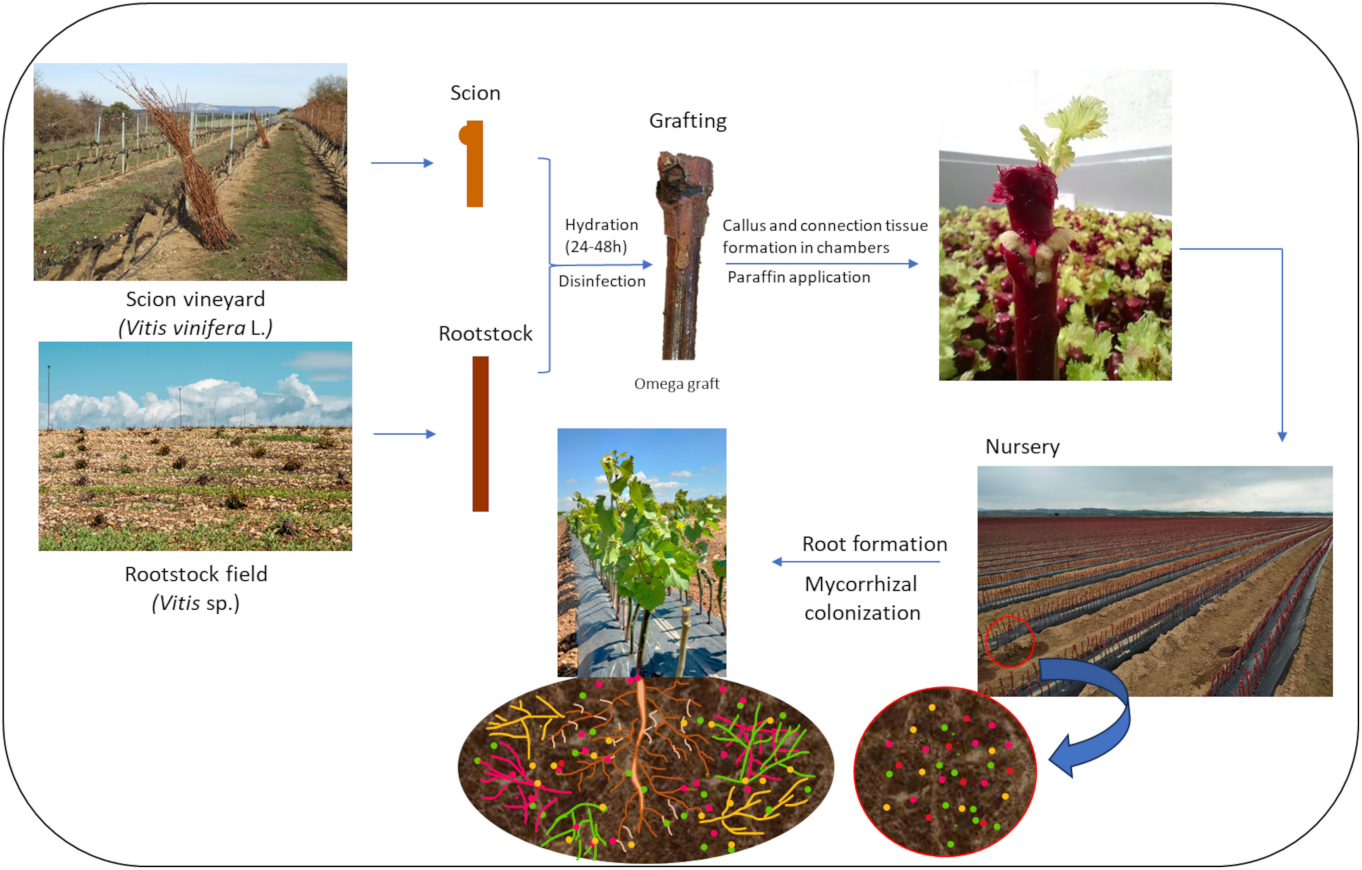
Schematic representation of the grapevine plant production system and mycorrhizal symbiosis establishment in nurseries. Wood from the scion vineyard and the rootstock field is collected, and dormant cuttings are prepared. Then, grafting is performed and callus and connection tissue formation takes place in chambers. Once the process is complete, unrooted grafted cuttings are planted in the nursery where natural mycorrhizal symbiosis occurs while roots are forming.

First contact between grapevine roots and AMF presumably occurs in nurseries, where every year worldwide between 800 million and 1.2 billion vines are produced though asexual propagation. Before the late 19th century, grapevine propagation was done in the field using cuttings from *Vitis vinifera* L. However, the accidental introduction of the phylloxera insect (*Daktulosphaira vitifoliae*) from North America to Europe revealed the susceptibility of *V. vinifera* roots to this pest. As a result, European grape varieties are now grafted onto rootstocks from North American *Vitis* species, which are more resistant to phylloxera. Nowadays, grafting is a common practice and is routinely used in more than 80% of vineyards worldwide (Ollat *et al*., 2016; Marín, 2022). Nurseries produce rooted and grafted plants through a relatively complex process shown in Fig. 2. The most critical points of the production process are (i) the preparation of dormant cuttings, (ii) bench grafting, (iii) layering for callus formation, (iv) planting of unrooted grafted cuttings in the nursery, and (v) uprooting and confection for selling (Aroca *et al*., 2010; Marín, 2022).

The rooting of vine plants grafted in nurseries begins from a dormant cane of the rootstock, which simultaneously strengthens the callus tissue at the graft junction. Roots formed in this vegetative propagation process are adventitious, that is roots that form from non-root tissues (Steffens and Rasmussen, 2016), and their formation requires a stimulatory effect such as a wound or cut, waterlogging, or the application of growth regulators (Tailor *et al*., 2022). Auxins are the main regulator of adventitious root development, their role being relatively well established (Daskalakis *et al*., 2019; Tailor *et al*., 2022; Boeno and Zuffellato-Ribas, 2023). However, several lesser-known factors are also involved in the process (Steffens and Rasmussen, 2016). Jiu *et al*. (2022) demonstrated that exogenous application of strigolactones markedly affects the root growth and development and alters the endogenous hormone levels. At this stage, the mycorrhizae should help the young plant to grow faster and be more tolerant to many stresses (see below, ‘Can arbuscular mycorrhizal fungi be used as natural biostimulants in viticulture?’). Early inoculation has been demonstrated to potentially facilitate the interaction between grapevine roots and these beneficial microorganisms to obtain a stable and functional symbiosis (Nerva *et al*., 2023). Unfortunately, few experiments have been conducted under real nursery conditions with grafted vines (Nikolaou *et al*., 2003), and experiments with young vines grown in pots might be biased.

Under these conditions, spontaneous mycorrhizal symbiosis depends solely on the presence of fungal structures in the nursery (Fig. 2), which can be limited by herbicide application. In adult vineyards, herbicides have detrimental effects on root colonization and propagule formation (Zaller *et al*., 2018), and favor a selection of herbicide-tolerant mycorrhizal species (Hage-Ahmed *et al*., 2019). There is evidence that herbicide residues may also modulate ecosystem-level outcomes via alteration of microbiomes (Ruuskanen *et al*., 2023), but little is known about the impact of growing conditions in the nurseries on the establishment of the mycorrhizal symbiosis, on the identity of *Glomeromycota* species selected, and on the effectiveness of the symbiotic relationship formed. Additionally, it is still unclear if the mycorrhizal symbiosis is effectively maintained in the transit from nursery to vineyards due to specific characteristics of nursery soils (i.e. crop rotation, chemical soil composition, and herbicide application) and to the fact that persistence of symbiosis is rootstock genotype dependent (Nerva *et al*., 2023) and could be enhanced by precropping with mycorrhizal plant donors (Nogales *et al*., 2009b).

## What we know about factors affecting mycorrhization in nurseries and vineyards

The effectiveness of mycorrhizal symbiosis in the vineyard, whether with naturally present or inoculated species, is complex because it depends not only on fungal identity (Bennett and Groten, 2022; Marro *et al*., 2022), but also on the high diversity of vine material compared with other cropping/agricultural systems (rootstocks, cultivars, and clones), edaphoclimatic conditions, and viticultural practices (Bettenfeld *et al.*, 2022). In the next subsections, the influence of these factors is presented in detail.

### Rootstock and scion genotypes

Both the scion and rootstock genotype of grafted grapevines influence the rhizosphere and root endophyte microbiomes (Noceto *et al*., 2024), but rootstocks have a greater impact (Lailheugue *et al*., 2024a, b). Thus, it was recently highlighted that the impact of the scion genotype on AMF community may be disputable, depending on the diversity metrics studied (i.e. α or β diversity) (Lailheugue *et al*., 2024b). Contrarily, it is widely accepted that rootstock genotype influences the distribution and composition of the soil microbial communities, including AMF (Marasco *et al*., 2018; Berlanas *et al*., 2019; Darriaut *et al*., 2022a; Lailheugue *et al*., 2024a, b; Noceto *et al*., 2024). *Glomeromycota* were often considered generalists with respect to the host, but there is increasing evidence of their preference for certain hosts (Holland *et al*., 2014). In fact, greater benefit derived from AMF symbiosis was observed when specific AMF species were used for certain rootstocks (Moukarzel *et al*., 2023), and a greater stimulation of plant growth and defense pathways was observed in them (Nerva *et al*., 2022, 2023). Differences in the AMF root community have also been found between genotypes in ungrafted plants (Moukarzel *et al*., 2021, 2022; Biasi *et al*., 2023), and can also occur between clones of one variety, as reported by Torres *et al*. (2016, 2018a, c). In addition to specific potential genetic affinities between the rootstock and the AMF, differences in rooting patterns can also contribute to the magnitude of mycorrhizal responsiveness of the host plant. Thus, less branched and coarser root systems, such as those from less stress-tolerant rootstocks, often benefit more from the relationship with the AMF (Newsham *et al*., 1995; Belew *et al*., 2010).

**Fig. 3.**
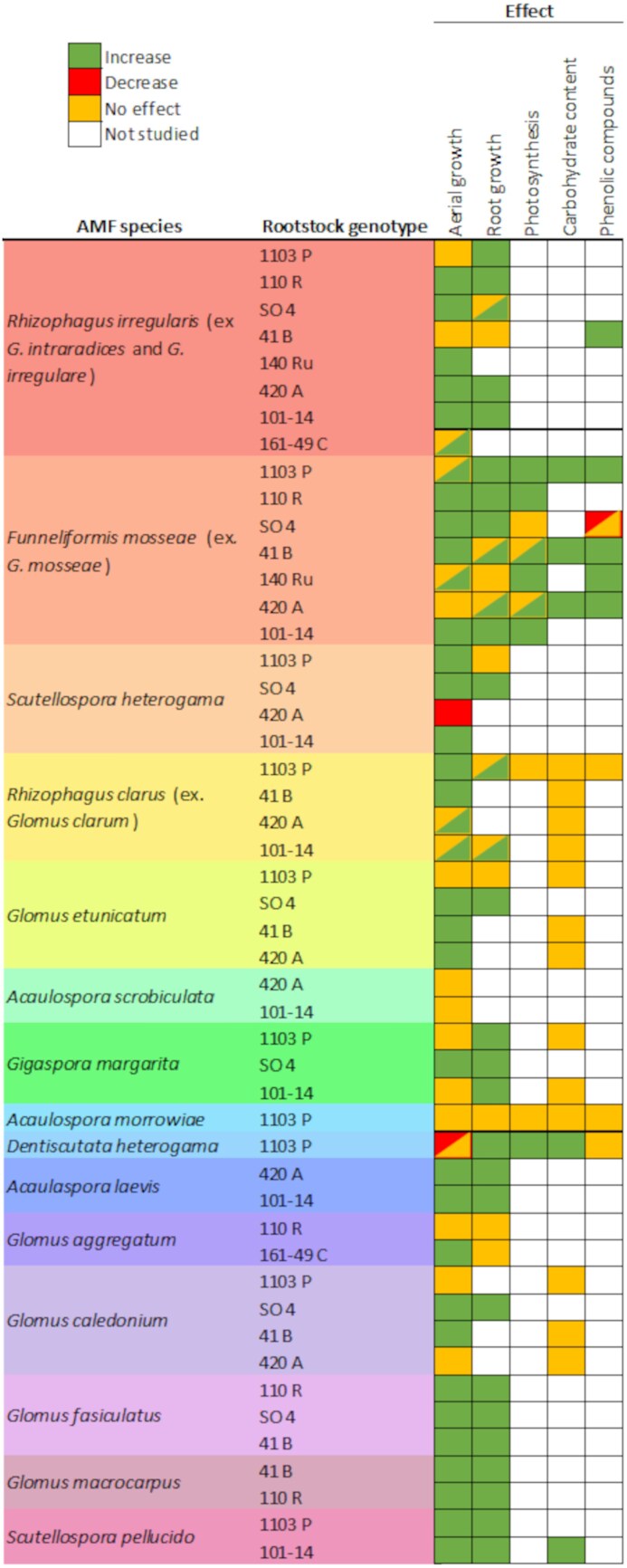
Effect of the arbuscular mycorrhizal fungal (AMF) species–rootstock interaction on grapevine physiology, growth, and metabolism under non-stressed conditions. Colored rectangles indicate increase (green), no effect (orange), decrease (red), or not studied (white) of the measured feature. Two-colored rectangles represent contrasting results reported in literature. (References used: Ravolanirina *et al.*, 1989; Karagiannidis *et al.*, 1995; Petgen, 1998; Linderman and Davis, 2001; Agostini and de Souza, 2003; Bavaresco *et al.*, 2003; Aguín *et al.*, 2004; Büttenbender and de Souza, 2005; Caglar and Bayrma, 2006; Bleach *et al.*, 2008; Nogales *et al.*, 2009a, b, 2019; Ozdemir *et al.*, 2010; Anzanello *et al.*, 2011; Cangahuala-Inocente *et al.*, 2011; Bruisson *et al.*, 2016; Rosa *et al.*, 2016.)

In Fig. 3, we provide a detailed compilation of the changes observed in grapevine physiology, growth, and metabolism depending on the combination of rootstock genotype and AMF species in earlier research. The more frequently observed effects of AMF inoculation were the enhancement of aerial and root growth with few exceptions. Regarding photosynthetic performance, the identity of the mycorrhizal fungus may affect gas exchange and carbohydrate accumulation to a greater extent than the rootstock genotype, whereas the inoculation of *F. mosseae* has a positive effect on grapevine metabolism in all the tested rootstocks (Fig. 3).

Altogether, the majority of these studies dealt with the effect on grapevine growth, but more research is needed to assess the impact of the interaction between AMF species and grapevine genotype (i.e. rootstock and scion genotypes) on other physiological processes. In fact, Lailheugue *et al*. (2024b) recently reported significant relationships between the root system microbiomes and the plant phenotype on its mineral status.

### Grapevine age

Vineyard age may affect native mycorrhizal richness and therefore colonization. Thus, mycorrhizal colonization was higher in younger grapevines compared with older vines (Betancur-Agudelo *et al*., 2021) and the number of AMF phylotypes or species was smaller in older vines (Schreiner and Mihara, 2009; Noceto *et al*., 2023). However, these differences in associated AMF communities regarding grapevine age could be restricted to the first two or three decades in the lifespan of a vineyard (Noceto *et al*., 2023) and might depend on soil chemical characteristics and grapevine response to different stresses (Betancur-Agudelo *et al.*, 2021). Finally, the higher biodiversity found in young vineyards (Schreiner and Mihara, 2009; Betancur-Agudelo *et al.*, 2021; Noceto *et al.*, 2023) need not be related to a higher efficiency of the mycorrhizal relationship. Previous studies showed that the positive effect of AMF on grapevine performance is not always enhanced when the inoculum consists of different mycorrhizal species compared with a single species inoculum (Nogales *et al*., 2009b).

### Effect of vineyard soil management on arbuscular mycorrhizal fungal communities

The effectiveness of the mutualistic relationship between AMF and grapevine may be influenced not only by biotic factors, such as the genotypes of grapevines and mycorrhizal fungi, but also by abiotic factors, including the growers’ management practices. Indigenous AMF populations are dependent on the soil characteristics, and therefore some cultural practices, such as mechanical and chemical tillage, or the presence of adventitious vegetation might modify naturally present AMF populations (Aguín *et al.*, 2004; Bowles *et al*., 2017; Ruuskanen *et al*., 2023).

For example, it is well known that tillage affects AMF diversity and colonization. Tillage diminished the diversity of AMF species (Oehl and Koch, 2018) and led to changes in the composition of AMF cohorts by decreasing their diversity (Kabir, 2005; Bowles *et al*., 2017). Furthermore, under those circumstances, species that grow rapidly or produce more vesicles and spores are likely to become dominant (Johnson and Pfleger, 1992; Douds *et al*., 1995; Jansa *et al*., 2003). In fact, some authors suggest that it would be advisable to minimize tillage, as intensive tillage is incompatible with long-term sustainable AM fungal populations (Holland *et al*., 2018).

Root infectivity, colonization, and the arbuscule density of AMF are also negatively impacted by the use of herbicides (i.e. glyphosate) (reviewed by Zaller *et al*., 2018; Ruuskanen *et al*., 2023). Soil AMF spore biomass, vesicles, and propagules were reduced after herbicide application, especially when glyphosate was leached toward the root zone by a simulated rainfall event (Zaller *et al*., 2014). Moreover, grapevines showed an altered nutrient composition in roots, leaves, grape juice, and xylem sap, especially when the herbicide used was glyphosate (Zaller *et al*., 2018), which might be hypothesized to be, at least in part, caused by the alterations in soil microbiology.

In contrast, the use of herbaceous plants as cover crops may favor the presence of AMF in vineyards, providing a wider spectrum of these fungi for colonizing grapevine roots (Radic *et al.*, 2012; Trouvelot *et al*., 2015). Furthermore, Capó-Bauçà *et al*. (2019) reported that after 7 years of using a permanent cover crop, an increase in spore number, microbial activity, and functional diversity of AMF was observed. However, the presence of cover crops does not always result in a rise of AMF abundance in grapevine roots, and some authors have reported that the presence of cover crops caused no changes in grapevine spontaneous mycorrhization (Baumgartner *et al*., 2010; Dangi and Wang, 2021). In any case, different species of cover crops have been identified as mycorrhizal donors when using them as an inoculant of mycorrhizal fungi in the field (Nogales *et al*., 2021). Nevertheless, as happens in other crop systems (e.g. maize), different cover crop species may promote certain AMF species that may be compatible or incompatible with grapevines. In fact, some studies reported higher diversity of AMF in roots of legumes, such as clover (i.e. specially Gigaspora phylotypes), than in wheat, and these variations can be explained by differences in root morphology (Higo *et al*., 2016). Altogether, this leads to the search for tools that strengthen the soil microbiome, and hence the plant-associated microbiome, through nutrient uptake and pathogen control, such as non-conventional soil management practices (i.e. cover crops) that can increase microbial diversity, presumably by increased organic matter, a rich source of grapevine microbial colonizers (reviewed by Darriaut *et al.*, 2022b). Therefore, a greater body of research is needed to fully understand the effects of vineyard soil management practices (especially, under the vines)—whether herbicide application, tillage, or cover cropping—on the interaction between grapevines and AMF communities under different edaphoclimatic conditions to the benefit of this relationship.

## Can arbuscular mycorrhizal fungi be used as natural biostimulants in viticulture?

### Evidence of the positive effects of inoculating with arbuscular mycorrhizal fungi

The pursuit of sustainable agriculture has boosted interest in the use of AMF as biostimulants in vineyards, concurrent with the development of commercial products based on these organisms. The long-term development of perennial plants and the exposure to natural AMF communities make it difficult to fully understand the true impact of AMF application on vine performance, while with annuals the effect is more apparent (Holland *et al*., 2018). Perennial plants can certainly benefit from the use of AMF biofertilizers, particularly when grown in environments with limited AMF. However, as broadly discussed by Bennett and Groten (2022), the cost and benefits of ‘artificially’ promoting AMF symbiosis need to be analysed considering the specific identities of the plant and fungus involved as well as the biotic and abiotic factors relevant to each situation.

**Fig. 4.**
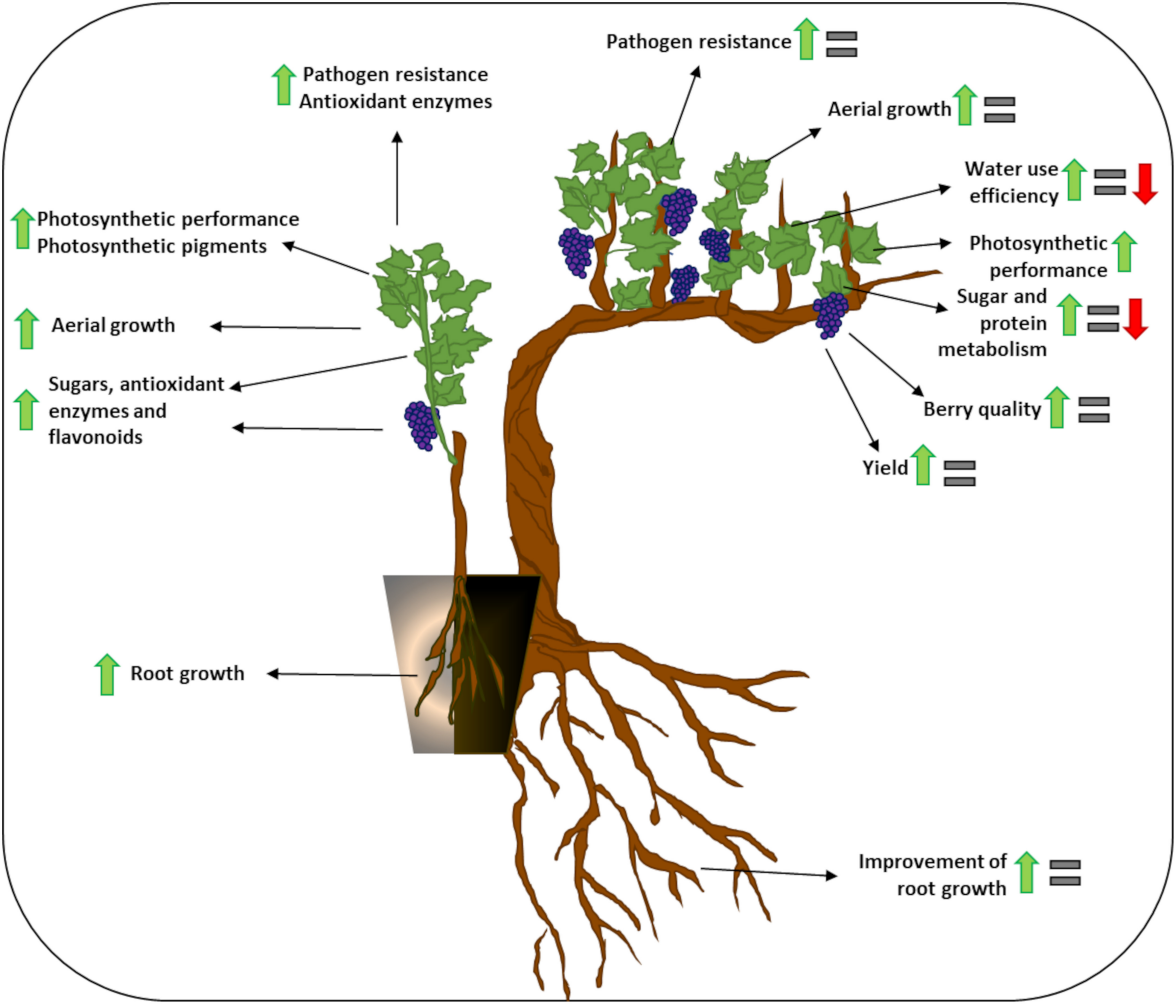
Schematic representation of the effect of arbuscular mycorrhizal fungal inoculation of grapevines under controlled (left) and field (right) conditions. A green arrow indicates enhancement, a red arrow indicates diminution, and the equals sign indicates no change of the measured feature when comparing inoculated versus non-inoculated vines.

Under controlled conditions, the inoculation with AMF is known to improve aerial and root growth, promote photosynthetic assimilation capacity, and increase photosynthetic pigments (i.e. chlorophylls), sugars, antioxidant enzymes, and flavonoids in leaves and berries (Fig. 4). These effects appear to be independent of the percentage of mycorrhization and were observed with different combinations of rootstock and AMF (Supplementary Table S1). However, a positive effect is not always achieved when inoculation is performed under field conditions (Fig. 4; Supplementary Table S2). Grapevines growing in the field face a combination of biotic and abiotic stresses difficult to reproduce in controlled environments. Therefore, biostimulants evaluated in controlled environments do not always perform as expected under field conditions, and some authors have emphasized the importance of adopting a ‘field to lab’ approach rather than a ‘lab to field’ approach (Rouphael *et al*., 2018),that is screening the biostimulant candidates under field conditions and, later, studying their mode of action under controlled conditions. In general terms, inocula containing multiple diverse AMF species may improve establishment of grapevine–AMF symbiosis and increase the potential for plant growth benefits (Supplementary Table S2), although this effect was not always observed (Ganugi *et al*., 2023).

In addition, the effectiveness of AMF biostimulants depends on the native microbiome and on edaphoclimatic factors. Despite the general idea of competition between the soil-native AMF and the AMF species of the inoculum, recent research revealed that inoculation of AMF in fields does not lead to a replacement of native root AMF communities. Thus, field inoculation allowed further colonization by other resident *Glomeraceae* and non-*Glomeraceae* AMF taxa (Nogales *et al*., 2021) and doubled the percentage of root colonization (Torres *et al*., 2021a). Additionally, AMF inoculation of grapevine roots has an impact on the vineyard soil microbiome leading to changes in the fungal and bacterial networks (Torres *et al.,* 2021b). Nevertheless, future research should also investigate how the inoculation of AMF might affect the activities of microbial enzymes involved in soil chemical cycling or community-level physiological profiling.

### The potential role of arbuscular mycorrhizal fungal inoculation in the context of climate change

**Fig. 5.**
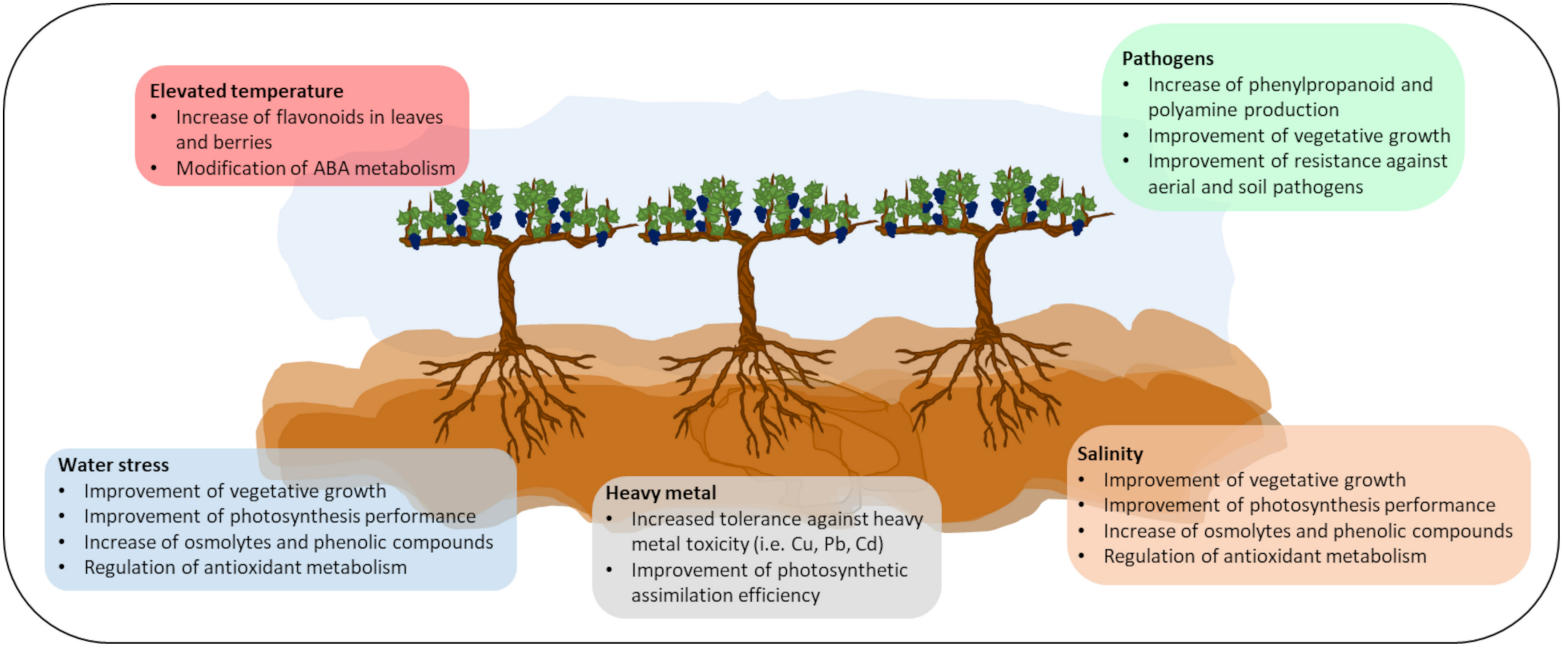
Main effects of inoculating grapevines subjected to abiotic and biotic stresses with arbuscular mycorrhizal fungi. ABA, abscisic acid. (References used: Karagiannidis and Nikolau, 2000; Nogales *et al.*, 2009a; Torres et al., 2015, 2016, 2018a, c, 2021a; Bruisson *et al.*, 2016; Upreti *et al.*, 2016; Vilvert *et al.*, 2017; Aslanpour *et al.*, 2019; Cetin *et al.*, 2019; Karimi and Noori, 2022; Biasi *et al.*, 2023; Brunetto *et al.*, 2023.)

The application of AMF as a biostimulant has been proposed to mitigate stresses associated with climate change (Torres *et al*., 2018b; Del Buono, 2021; Aguilera *et al*., 2022) given the ability of AMF to enhance grapevine response against abiotic and biotic stresses (Supplementary Table S3). Climate change is expected to have a detrimental effect on grapevine cultivation given the rise in temperature and the increased variability in precipitation, which will account for intermittent heat peaks, seasonal drought, and increased evaporation (Jones *et al*., 2005; Hannah *et al*., 2013). In arid and semi-arid regions, higher evaporation and decreased water availability can additionally lead to soil salinity issues and heavy metal toxicity (Ullah *et al*., 2021). Climate change has significant impacts on grapevine as a host plant for phytophagous insects, and on trophic interactions in the vineyard by modifying their phenology or distribution ranges (Reineke and Thiéry, 2016). The main effects of AMF inoculation reported in earlier research in grapevines facing abiotic and biotic stresses are presented in Fig. 5.

Under elevated temperatures, inoculation with AMF has been shown to promote flavonoid accumulation in leaves and berries (Torres *et al*., 2015, 2016; Goicoechea *et al.*, 2023), presumably by shifts in abscisic acid (ABA) metabolism induced by AMF (Torres *et al*., 2018c). AMF inoculation tended to accumulate ABA-glucose ester and 7′-hydroxy-ABA to the detriment of phaseic acid (Torres *et al*., 2018c). AMF inoculation could also help sustain grape growth under thermal stress conditions, especially after heat shocks (Nogales *et al*., 2020). Furthermore, AMF improve tolerance to water stress, photosynthetic capacity, and vegetative growth under water deficit conditions by increasing the content of phenolic compounds and soluble proteins and improving antioxidant enzymes (Cetin *et al*., 2019; Torres *et al*., 2021a). This improvement in vine water status may be related to the increased presence of mycorrhizal structures (arbuscules and vesicles), which are known to improve water uptake and nutrient storage (Kakouridis *et al*., 2022; Biasi *et al*., 2023). Therefore, grapevines compensate for a lower density of fine roots by stimulating the colonization of AMF (Schreiner, 2007) which allows the plant to absorb water more efficiently, enabling the grapevine to cope with water stress. Additionally, AMF-inoculated grapevines increase stomatal conductance and transpiration rate and decrease the concentration of intercellular CO_2_ making the plant more resistant to abiotic stresses (Ye *et al*., 2022). Nevertheless, the majority of existing studies have been carried out with pot-based experiments (Supplementary Table S3), underscoring the need for additional research under field conditions, which presents challenges due to the complexities involved in conducting controlled high-temperature experiments in field settings.

Another climate change-derived factor is the increment of salinity, and the role of AMF in increasing salinity tolerance in grapevines was recently pointed out. At low salinity levels, AMF increased antioxidant enzyme activities and accumulated metabolites such as soluble sugars, proteins, proline, phenols, and flavonoids in grapevines (Karimi and Noori, 2022), while at medium to high levels there was an induction of mechanisms related to salinity (increased K/Na ratio) and resistance to abiotic stress (polyamine production) in plants (Upreti *et al*., 2016). AMF symbiosis has also been associated with a higher plant tolerance to heavy metal toxicity (Fig. 5), such as Cu (Brunetto *et al*., 2023) or Cd and Pb (Karagiannidis and Nikolau, 2000). However, contrasting results were obtained when there were high concentrations of nutrients in the soil (Supplementary Table S3). In addition, organic vineyard management in relation to Cu application requires further research into the toxicity threshold for this metal and the response of the symbiosis to it.

It is well established that inoculation with AMF acts as an elicitor of vine defenses, and its role in grapevine resistance to grey mold, downy mildew, and Armillaria root rot diseases has been previously studied (Supplementary Table S3). At the biochemical level, AMF promote the metabolism of polyamines (Nogales *et al*., 2009a), hydrolytic enzymes (Costa *et al*., 2010), and pathogen-resistant proteins (Vilvert *et al*., 2017), which accounted for a higher resistance to these pathogens. Furthermore, Bruisson *et al.* (2016) demonstrated that AMF induce the expression of phenylpropanoid biosynthesis and stilbenoid key gene expression in grapevine leaves in response to downy mildew and grey mold infection. However, little is yet known about the underlining mechanisms enhanced by AMF that protect vines against these pathogens.

Finally, it is worth mentioning that commercial inocula frequently contain a mix of AMF and plant growth promoting (PGP) microbes, increasing the benefits of inoculation, although the combined effects are still under consideration. Thus, Noceto *et al*. (2021) reviewed that co-inoculation of AMF and PGP microbes enhanced crop yield and fruit quality by modifying sugars, acids, carotenoids, flavonoids, and antioxidant compounds in some crops (i.e. strawberry and tomato), but these effects differed according to the identity of the partners (i.e. AMF and PGP bacterial species). Along these lines, Ujvári *et al*. (2021) highlighted that AMF could serve as effective tools for introducing beneficial root endophytes in sustainable and organic agriculture, where the functioning of these complex associations may play a vital role in crop production.

## Final remarks

**Fig. 6.**
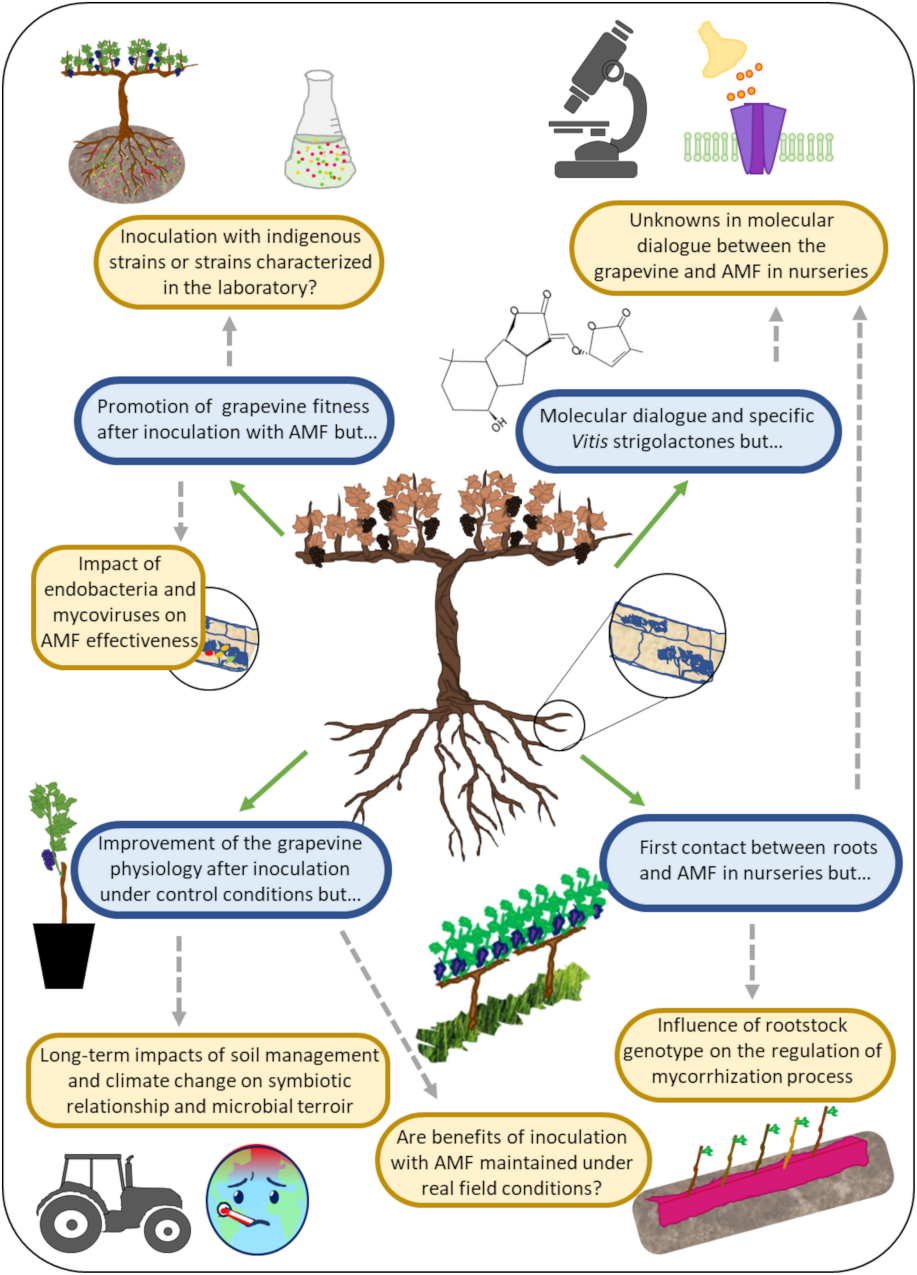
Overview of the main knowns (blue panels) and unknowns (yellow panels) of research on grapevine and arbuscular mycorrhizal fungi (AMF).

The role of AMF in viticulture has been broadly discussed in the last two decades. Nevertheless, current challenges make it imperative to reach a better understanding not only of the mycorrhizal symbiosis process from the nursery to the field, but also of how biotic and abiotic factors affect its effectiveness. In this sense, Fig. 6 is a resumé of current knowledge about AMF and grapevines and the remaining unanswered questions discussed in this review.

Further research on how the symbiotic relationship between AMF and grapevines is built in nurseries and how it evolves after transplanting into the vineyard is needed. In particular, little is known about the molecular dialogue between the grapevine and the fungus during this stage and how the rootstock genotype may influence the regulation behind the process of mycorrhization. In this regard, the lack of specificity of the strigolactone signal (Clark and Bennett, 2023), the recent discovery of a specific *Vitis* non-canonical strigolactone (Lailheugue *et al*., 2023), and the subsequent exudation of fungal and plant molecules opens a field of research into how they work in order to efficiently apply AMF inocula, and into the place of microbial endophytes/partners in the vine holobiont. Additionally, the study of plasticity of PGP microbes in promoting tolerance to abiotic stresses and their impact on the interactions between rootstocks, scions, and their combinations at the field level highlights the importance of incorporating the microbiome in grapevine breeding programs (reviewed by Darriaut *et al*., 2022b). Finally, it would be interesting to investigate if the fungal exudates are impacted by the endobacteria because a change in these exudates could modify the rhizosphere ecosystem, as fungal exudate-consuming bacteria live on the surface of fungal hyphae and spores (Verhage, 2021).

On the other hand, in a context where grape growers are moving towards more sustainable soil management practices (Lazcano *et al*., 2020; Ochoa-Hueso *et al.,* 2024), it is worth focusing on how these operations can help AMF activity and provide ecosystemic services to the vineyard. There is still little research on the effect of cover crops, minimum tillage, mulching, and other soil regenerative practices on these interactions. Furthermore, it is essential to understand both the short-term effects (1–2 years) and the longer-term impacts (more than 5 years) of soil management practices on AMF activity and composition, as well as how these factors influence the microbial terroir. Although AMF inoculation may be beneficial, it is crucial to keep in mind the need to protect mycorrhizal communities in the soil to provide durable and resilient plant–AMF associations. This work highlights the eventual potential of using AMF as a biostimulant under climate change conditions. Thus, AMF inoculation might promote greater tolerance to elevated temperatures, water and salinity stresses, heavy metal toxicity, and pathogens. However, as this work highlights, evidence has been mainly obtained from greenhouse experiments that have some limitations, and further studies carried out under field conditions are advisable to unravel if the benefits of AMF inoculation of grapevines are maintained under real field conditions and condition their response to stresses.

In conclusion, while much remains to be discovered about the interaction between mycorrhizae and grapevines, addressing these ‘known unknowns’ presents a valuable opportunity for research. Such efforts can provide scientists and wine growers with deeper insights and innovative solutions to the challenges they face in viticulture.

## Supplementary data

The following supplementary data are available at *JXB* online.

Table S1. Effects of arbuscular mycorrhizal fungi–grapevine symbiosis on experimental designs conducted with potted vines.

Table S2. Effects of arbuscular mycorrhizal fungi–grapevine symbiosis on experimental designs conducted with field-grown vines.

Table S3. Effects of arbuscular mycorrhizal fungal inoculation of vineyards subjected to biotic and abiotic stresses.

eraf081_suppl_Supplementary_Tables
