# Supplementary material for: Mycorrhizae and grapevines: the known unknowns of their interaction for wine growers’ challenges
Source: J Exp Bot. 2025 Mar 11;76(11):3001–15. doi: 10.1093/jxb/eraf081 (PMC12321743; doi:10.1093/jxb/eraf081)
Supplement: eraf081_suppl_Supplementary_Tables [file eraf081_suppl_supplementary_tables.pdf]

Supplementary Table S1. Effects of arbuscular mycorrhizal fungi (AMF)-grapevine symbiosis on experimental designs conducted with potted vines.

AMF species	Rootstock genotype	% root length colonized	Growth conditions (pot characteristics: substrate & irrigation)	Effects	Reference
*Commercial AMF inoculum (R. irregularis** and *F. mosseae*)*	SO 4, 1103 P	-	2 L pots: substrate not sterilized (vineyard soil: sphagnum peat (8:2, v:v)) and daily watered	Improved aerial and root growth and enhanced stress tolerance (ABA production in roots) and pathogen resistance (stilbene production)	Nerva *et al*., (2022)
Soil with AMF families (Acaulosporaceae, Glomeraceae, Claroideoglomeraceae, Gigasporaceae, Paraglomeraceae, Archaeosporaceae, Ambisporaceae) + inoculum of *R. clarus**	1103 P	≈50%-70%[a]	Pots: vineyard soil not sterilized and without irrigation	Improved aerial and root growth and better photosynthetic assimilation capacity.	Betancur-Agudelo *et al*., (2020)
F. mosseae	Dog ridge	46.2%[b]	36-cm diameter and 41-cm height pots: substrate pre-sterilized (red sandy loam soil: sand (2:1, w:w and daily watered	Improved aerial and root growth and increased the chlorophyll, abscisic acid (ABA), cytokinin and polyamine (spermidine and spermine) contents in the leaves	Upreti *et al*., (2016)
Mix of AMF (*G. intraradices*, *G. aggregatum*, *F. mosseae, R. clarus*, *G. monosporus*, *G. deserticola*, *G. brasilianum*, *G. etunicatum* and *G. margarita*)	5 BB, 41 B, 110 R	-	12 × 25 cm polyethylene sacks: substrate (perlite: turf (1:1, v:v))	Improved root growth and aerial part length. Enhanced the chlorophyll, soluble sugar and phenolic compound content.	Cetin *et al*., (2014)
R. irregularis or F. mosseae	1103 P	63%[c] and 70%[c], respectively	600 mL pots: substrate sterilized (sphagnum peat: perlite (2:1, v:v)) and daily watered	Improved root growth	Nogales *et al*., (2019)
Inoculum with species of Glomeraceae and Ambisporaceae family ('home' and 'away' communities)	101-14, 5 C, Schwarzmann	-	20 × 30 cm planting bags: substrate (20% silica sand and 80% low P potting mix containing fertilizers: Osmocote, horticultural lime, Micromax, and Hydraflo) and manual irrigation	Improved root and shoot weight with their "home" AMF communities	Moukarzel *et al*., (2023)
F. mosseae	Self-rooted Sultana	-	Pots: substrate sterilized (perlite: coco peat (1:1, v:v) and irrigated each three days	Improved aerial and root growth and enhanced SPAD index, relative water content and catalase activity	Karimi and Noori, (2022)
G. margarita, G. margarita + D. heterogama, D. heterogama, R. irregularis, A. colombiana, A. colombiana + A. scrobiculata, A. scrobiculata	IAC-766 C	≈30%-53%[c]	Commercial substrate (biostabilized pine bark: washed sand (1:1)) and manual irrigation each two days	Improved aerial and root growth and root length showing differences between the AMF species and combinations	Vilcatoma-Medina *et al*., (2023)
R. irregularis	SO 4	62.90%[b]	1 L pots: clay-loam soil: soil fertilizer conditioner (1:1, v:v); daily watered to field capacity and 10 ml of nutrient solution per week.	Improved aerial and root growth	Hao *et al*., (2012)
R. irregularis	Riparia Gloire, Schwarzman, Salt Creek	-	7.6 L pots: Expanded clay (Turface; Profile products LCC) and daily watered (60 mL per pot)	Increased biomass production only in the greenhouse	Rosa *et al*., (2020)
Commercial AMF inoculum (R. irregularis, G. aggregatum, F. mosseae, R. clarus, G. monosporus, G. deserticola, G. brasilianum, G. etunicatum and G. margarita)	5 BB or 110 R	-	Pots: substrate (perlite: turf (1:1, v:v) and regularly irrigated (50 mL per week)	Improved aerial and root growth. Enhanced antioxidant enzymes (CAT and APX) for 5BB and Changes in antioxidant enzymes (enhanced APX and decreased SOD) for 110R	Cetin *et al*., (2019)
Commercial AMF inoculum (*R. irregularis*)	Riparia Gloire, 101-14, SO 4	62.90%[a]	2 L pots: substrate sterilized (sand: Turface (1:1, w:w)) and irrigated each two days (100 mL per pot)	Only Improved SPAD for SO4, but not photosynthesis	Holland *et al*., (2018a)

R. irregularis	110 R or SO 4	-	400 mL pots: substrate sterilized (organic substrate (Germina Plant, Turfa Fértil®): soil: sand (2:1:2, v:v:v)) and daily watered with distilled water and nutrient solution once a week.	Improved root growth	Vilvert *et al*., (2017)
AMF mix (S. deserticola, F. mosseae, R. irregularis, R. clarus and G. aggregatum) + a mixture of rhizobacteria belonging to the Bacillus and Paenibacillus	Self-rooted Tempranillo	31.50%[c]	6.5 L pots: substrate sterilized (vermiculite: sand: light peat (2.5:2.5:1, v:v:v) and watered twice per day (140 mL of nutrient solution per pot)	Enhanced phenolic, aminoacidic and aromatic precursor contents in berries	Torres *et al*., (2019)

* *R. irregularis* (formerly *G. irregulare* and *G. intraradices*); *F. mosseae* (formerly *G. mosseae*); *R. clarus* (formerly *G. clarum*).

[a,b,c] Methods of colonization determination: a) Magnified intersection method (McGonigle et al., 1990), b) Root segment estimation method (Hayman, 1970; Trouvelot et al., 1986), c) Grid-line intersect method (Giovanetti & Mosse, 1980).

Supplementary Table S2. Effects of arbuscular mycorrhizal fungi (AMF)-grapevine symbiosis on experimental designs conducted with field-grown vines.

AMF species	Rootstock genotype	Grafted	% root length colonized	Growth conditions (Irrigation & Fertilization)	Effects	Year of vines	Experiment duration (years)	Reference
R. irregularis	Riparia Gloire	No	-	Drip irrigation	No difference	2	2	Rosa *et al.* (2020)
	Schwarzmann		-		No difference			
	Salt Creek		-		Increased belowground biomass			
R. irregularis	140 Ru	Cabernet Sauvignon	-	Not specified	Improved growth in both years in a vineyard with 10 years of tillage	1	2	Nogales *et al.*, (2009b)
	161-49 C		-		Improved growth in the first year but not in the second year in a vineyard with 10 years of tillage			
R. irregularis and *F. mosseae*	1103 P	Glera	-	Watered 3 times per week for 2 h (6 L h^{-1} per plant) over the vegetative season with a subsoil irrigation system.	Improved the water use efficiency, decreased the transpiration and enhanced pathogen resistance (viniferin production)	2-3	2	Nerva *et al.*, (2023)
	SO 4		-		Improved the water use efficiency, decreased the transpiration and enhanced the leaf IAA concentration.			
Commercial AMF inoculum (R. irregularis, *F. mosseae* and G. *etunicatum)*	SO 4	Solaris, Regent	-	Not irrigated and not fertilized	No differences on growth, yielding and healthiness	1	7	Lisek *et al.*, (2016)
Mycorrhizal treatment (N.D., application of mycorrhizal roots)	Dog Ridge	Thompson Seedless	-	Not specified	Improved aerial growth, the quality of fruits and the yield. Enhanced the photosynthesis and increased the protein and phenols content in the leaves but decreased soluble sugars.	8	2	Somkuwar *et al.*, (2014)
Commercial AMF inoculm (*F. mosseae*, *F. monosporum*, *R. clarus*, *R. aggregatum*, *R. irregularis*, *S. deserticola*, *C. etunicatum*, *G. margarita* and *P. brasilianum*)	3309 C	Riesling, Pinot noir	63%[a]	Not specified	Improved root growth	>20	1	Berdeja *et al.*, (2023)
	SO 4	Riesling	64%[a]	Irrigated	Improved root growth			
Commercial AMF inoculum (*R. irregularis*, *F. mosseae* and *C. etunicatum*)	SO 4	Cabernet Sauvignon	79-82%[a]	Not irrigated and not fertilized	Improved leaf gas exchange parameters and higher yield parameters. Higher total flavan-3-ols, total anthocyanins, and total polyphenols in berry skin.	>10	2	Karoglan *et al.*, (2021)
Commercial AMF inoculum (*R. irregularis*, *F. mosseae* and *C. etunicatum)*	SO 4	Pinot Noir	-	Fertilized only in the first year with a multi-component fertilizer Suprofos 25 NPK (Ca, Mg, S) 5:10:25 (2.5:2:13) at the dose of 400 kg/ha.	Increased the intensity of CO_2 assimilation and transpiration and reduced the efficiency of photosynthetic water use and increased stomatal conductance for water in the grapevines tested.	2	2	Mikiciuk *et al.*, (2018)
	5 BB	Regent	-					
	125 AA	Rondo	-					

* *R. irregularis* (formerly *G. irregulare* and *G. intraradices*); *F. mosseae* (formerly *G. mosseae*); *R. clarus* (formerly *G. clarum*).

[a] Method of colonization determination: Magnified intersection method (McGonigle et al., 1990)

Supplementary Table S3. Effects of AMF inoculation of vineyards subjected to biotic and abiotic stresses.

AMF species inoculated	Plant material and grown conditions	Experiment	% root length colonized	Stress	Effects	Reference
**Elevated temperature**						
R. irregularis	Tempranillo self-rooted	Cuttings grown in pots with substrate	15%[d]	Elevated temperature (+4°C)	Improvement of the phenolic accumulation in berries	Torres *et al.*, (2016)
R. irregularis	Tempranillo self-rooted	Cuttings grown in pots with substrate	15%[d]	Elevated temperature (+4°C)	Increased flavonoid contents in leaves	Torres *et al.*, (2015)
R. irregularis	Tempranillo self-rooted	Cuttings grown in pots with substrate	15%[d]	Elevated temperature (+4°C) + deficit irrigation	Improved berry quality, especially under late deficit irrigation and elevated temperature phenolic accumulation in berries	Torres *et al.*, (2018a)
AMF mix (*S. deserticola*, *F. mosseae*, *R. irregularis*, *R. clarus* and *G. aggregatum*) + a mixture of rhizobacteria belonging to the Bacillus and Paenibacillus	Tempranillo self-rooted	Cuttings grown in pots with substrate	29.4-62.3%[c]	Elevated temperature (+4°C) + deficit irrigation	AMF increased berry anthocyanins and modulated ABA metabolism, leading to higher ABA-GE and 7'OH-ABA and lower phaseic acid (PA) in berries compared to non-mycorrhizal vines	Torres *et al.*, (2018c)
**Water stress**						
G. fasiculatu, R. intraradices or F. mosseae	White currant	Cuttings grown in pots with soil + substrate	-	Drought stress	Enhanced water stress tolerance. Improved photosynthesis index and sub-stomatal CO_2 and decreased transpiration rate and leaf area temperature. Decreased transpiration rate and leaf area temperature and enhanced sub-stomatal CO_2	Aslanpour *et al.*, (2019)
Commercial AMF inoculum (*R. irregularis, G. aggregatum F. mosseae, R. clarus, G. monosporus, G. deserticola, G. brasilianum, G. etunicatum and G. margarita)*	5 BB or 110 R	Cuttings grown in pots with substrate	-	Drought stress	Enhanced water stress tolerance. Improved aerial and root growth, proline, total phenolic and soluble protein content and reduced H_2O_2 and MDA content. Improved antioxidant enzymes (SOD, and APX) and decreased CAT and Improved aerial growth.	Cetin *et al.*, (2019)
Commercial AMF inoculum *R. irregularis*, *F. mosseae*, *G. aggregatum* and *G. etunicatum*	3309 C	Field experiment in a vineyard of Merlot	24.09%[d]	Half irrigated	Improved vegetative growth, photosynthetic activity, and water status and involved in the regulation of anthocyanin and flavonol metabolism	Torres *et al.*, (2021a)
Natural soil AMF community	420 A, Aleatico self-rooted	Field experiment in a vineyard of 'Aleatico' variety	77.30-86.7%[b]	Drought/heat stress	Increased AMF colonization strategies (greater abundance of arbuscules and mycorrhizal colonization potential) linked to the improved uptake and transport of water and Increased leaf resilience traits (high chlorophyll content and stomatal conductance values) and larger AMF storage organs (vesicles).	Biasi *et al.*, (2023)
Commercial AMF inoculum *(G. coronatum, G. caledonium, F. mosseae, G. viscosum* and *R. irregularis).*	1103 P	Field experiment of rootstock cuttings	-	Drought stress	Enhance water stress resistance and improved vegetative growth	Cardinale *et al.*, (2022)
**Salinity stress**						

F. mosseae	Sultana self-rooted	Cuttings grown in pots with substrate	-	Salinity (75 mM)	Enhanced salinity tolerance (increased antioxidant enzymes activities, soluble sugars and proteins, proline, phenol and flavonoid content). Improved aerial and root growth, enhanced SPAD index, and relative water content.	Karimi and Noori, (2022)
F. mosseae	Dog Ridge	Cuttings grown in pots with sterilized soil	28%[b] and 18%[b], respectively	Medium (150 mM) to high (250 mM) salinity	Enhanced salinity stress resistance (increased K/Na ratio) and stress abiotic resistance (polyamine production). Improved aerial and root growth, the water and osmotic potential, the photosynthesis rate and the acid abscisic and cytokinins contents in leaves.	Upreti *et al.*, (2016)
**Heavy metal toxicity**						
R. irregularis or F. mosseae	1103 P	Grafted plants of Touriga Nacional grown in pots with no sterilized soil	52-59%[c]	Cu	Avoided Mn increased in leaves, No enhanced Cu stress tolerance. Sustained leaf Fe levels but decreased N and P contents	Nogales *et al.*, (2019)
D. heterogama, R. clarus or R. irregularis	1103 P	Field experiment of rootstock cuttings	-	Cu	Decreased height, but enhanced Cu tolerance (lower Cu levels in leaves)	Brunetto et al., (2023)
Soil with families (Glomeraceae, Archaeosporaceae and Ambisporaceae) + inoculum of *AMF species*	1103 P	Grafted plants of Kazaki grown in pots with no sterilized soil	22-30%[a]	Cu	Improved C absorption and decreased photosynthetic activity. (Mycorrhizal inoculation did not affect grapevine growth because high concentrations of nutrients in the soil inhibit mycorrhizal colonization) but improved photosynthetic assimilation efficiency	Betancur-Agudelo *et al.*, (2020)
AMF soil mix (*G. macrocarpum*, *F. mosseae*, *G. fasciculatum*, *G. gigantea*, *A. laevis* and *Scutellospora.* sp)	110 R	Grafted plants of Kazaki grown in pots with no sterilized soil	26,30%[c]	Pb	Improved plant growth and enhanced Brix value, titratable acidity and decreased Pb leaf and berry content	Karagiannidis and Nikolau, (2000)
AMF soil mix (*G. macrocarpum*, *F. mosseae*, *G. fasciculatum*, *G. gigantea*, *A. laevis* and *Scutellospora.* sp)	110 R	Grafted plants of Kazaki grown in pots with no sterilized soil	44,70%[c]	Cd	Improved plant growth and decreased Cd leaf and berry content	
**Resistance against pathogens**						
R. irregularis	41 B	Cuttings grown in pots with substrate	-	*Infection of Plasmopara viticola* and *Botrytis cinerea*	Enhanced phenyl propanoid genes and defensive response (production of stilbenes) to the aerial pathogens *P. viticola* and *B. cinerea*	Bruisson *et al.*, (2016)
R. irregularis	110 R	Cuttings grown in pots with sterilized soil + substrate	76%[c]	Infection of *Armillaria mellea*	Improved aerial and root growth and increased tolerance against *A. mellea* (polyamine production at the beginning of the pathogenic infection)	Nogales *et al.*, (2009a)
R. irregularis	SO 4	Cuttings grown in pots with substrate	65.20%[b]	*Infection of Xiphinema index*	Reduced development of the nematode and improved aerial and root growth.	Hao *et al.*, (2012)
R. irregularis	SO 4	Micropropagated plants grown in pots with substrate	66%[b]	*Infection of Fusarium oxysporum*	Enhanced resistance against *F. oxysporum* wilt and improved growth and increased expression of hydrolytic enzymes chitinases and β-1,3-glucanases.	Costa *et al.*, (2010)
R. irregularis	110 R or SO 4	Cuttings grown in pots with substrate	-	*Infection of Fusarium oxysporum*	Enhanced *F. oxysporum* resistance and improved root growth	Vilvert *et al.*, (2017)
R. irregularis	110 R	In vitro propagation	10%[c]	Infection of *Armillaria mellea*	Alleviated necrosis and growth decrease in root and improved aerial growth	Nogales *et al.*, (2010)
Inoculum with species of Glomeraceae and Ambisporaceae	101-14, 5 C and Schwarzmann	Cuttings grown in pots with substrate	-		Reduced the incidence and severity of black foot disease and did not reduce their growth.	Moukarzel *et al.*, (2022)

family ('home' and 'away' communities)						
S. calospora, F. mosseae R. irregularis and *R.* sp	420 A, 101-14, 110 R, 1103 P, 3309 C or Pinot noir self-rooted	Green growing vines grown in pots (fumigated soil)	82- 95%[a]	Infection of *Mesocriconema xenoplax*	Reductions in both fine root production and AMF colonization due to the ring nematode, especially in less resistant rootstocks.	Schreiner *et al.*, (2012)
Commercial AMF inoculum *(R. irregularis)*	Riparia Gloire	Cuttings grown in pots with substrate	-	Infection of *Ilyonectria liriodendra*	Increased the abundance of *Ilyonectria* (Black foot disease)	Holland *et al.*, (2019)

* *R. irregularis* (formerly *G. irregulare* and *G. intraradices*); *F. mosseae* (formerly *G. mosseae*); *R. clarus* (formerly *G. clarum*).

[a,b,c,d] Methods of colonization determination: a) Magnified intersection method (McGonigle et al., 1990; Schreiner 2003), b) Root segment estimation method (Trouvelot et al., 1986; Hayman, 1970), c) Grid-line intersect method (Giovanetti & Mosse, 1980), d) Intensity of mycorrhizal colonization (Torres et al., 2015).

Supplementary references

Giovannetti M, Mosse B. 1980. An evaluation of techniques for measuring vesicular arbuscular mycorrhizal infection in roots. New Phytologyst 84(3), 489–500. https://doi.org/10.1111/j.1469-8137.1980.tb04556.x.

Hayman DS. 1970. Endogone spore numbers in soil and vesicular–arbuscular mycorrhiza in wheat as influenced by season and soil treatment. Transactions of British Mycological Society 54(1), 53– 63. https://doi.org/10.1016/S0007-1536(70)80123-1.

McGonigle TP, Miller MH, Evans DG, Fairchild GL, Swan JA. 1990. A new method which gives an objective measure of colonization of roots by vesicular–arbuscular mycorrhizal fungi. New Phytologyst 115(3), 495–501. https://doi.org/10.1111/j.1469-8137.1990.tb00476.x

Trouvelot A, Kough JL, Gianinazzi-Pearson V. 1986. Estimation of VA mycorhizal infection levels. Research for method having a functional significance. In: Gianinazzi-Pearson V, Gianinazzi S, eds. Physiological and genetical aspects of mycorrhizae. First European Symposium on Mycorrhizae. Dijon, France, July 1–5, 1985 Proceedings. Paris, Institut National de la Recherche Agronomique, 217–221.

Torres N, Goicoechea N, Antolín MC. 2015. Antioxidant properties of leaves from different accessions of grapevine (Vitis vinifera L.) cv. Tempranillo after applying biotic and/or environmental modulator factors. Industrial Crops and Products 76, 77-85. https://doi.org/10.1016/j.indcrop.2015.03.093.

Schreiner RP. 2003. Mycorrhizal Colonization of Grapevine Rootstocks under Field Conditions. American Journal of Enology and viticulture 54(3), 143-159. https://doi.org/10.5344/ajev.2003.54.3.143
